# Cost-effectiveness analysis of olaparib as maintenance therapy in patients with platinum-sensitive relapsed ovarian cancer and a BRCA1/2 mutation in china

**DOI:** 10.3389/fphar.2022.818579

**Published:** 2022-08-12

**Authors:** Yamin Shu, Yanxin Liu, Xucheng He, Yufeng Ding, Qilin Zhang

**Affiliations:** ^1^ Department of Pharmacy, Tongji Hospital, Tongji Medical College, Huazhong University of Science and Technology, Wuhan, China; ^2^ Department of Pharmacy, Pengzhou People’s Hospital, Pengzhou, China; ^3^ Department of Pharmacy, Pengzhou Second People’s Hospital, Pengzhou, China; ^4^ Department of Pharmacy, Union Hospital, Tongji Medical College, Huazhong University of Science and Technology, Wuhan, China

**Keywords:** cost-effectiveness analysis, SOLO2 trial, ovarian cancer, olaparib, BRCA1/2 mutation

## Abstract

**Objective:** The aim of this study was to investigate the cost-effectiveness of olaparib as the maintenance therapy in patients with platinum-sensitive relapsed ovarian cancer and a BRCA1/2 mutation in China.

**Methods:** A Markov model was developed to simulate the clinical course of typical patients with ovarian cancer in the SOLO2 trial. The Weibull survival model was employed to fit the Kaplan–Meier progression-free survival and overall survival probabilities of the olaparib and placebo strategies, respectively. The clinical and direct costs data were derived from randomized clinical trials and published reports. Quality-adjusted life-years (QALYs) and incremental cost-effectiveness ratios (ICERs) were estimated over a 10-year lifetime horizon. Meanwhile, one-way and probabilistic sensitivity analyses were used to explore the impact of uncertainty on the model’s outcomes.

**Results:** Overall, the incremental effectiveness and cost of olaparib versus placebo were 0.56 QALYs and $43,292.92, respectively, resulting in an ICER of $77,620.56/QALY, higher than the willingness-to-pay (WTP) threshold of China ($31,498.70/QALY). The results were sensitive to the cost of olaparib and utility of PFS. Scenario analyses suggested that when the cost of olaparib was reduced by 60%, ICER decreased to $30,611.52/QALY, lower than the WTP threshold of China.

**Conclusion:** The findings from the present analysis suggest that olaparib with a 60% discount as maintenance therapy might be cost effective in patients with platinum-sensitive relapsed ovarian cancer and a BRCA1/2 mutation in China.

## Introduction

Ovarian cancer is one of the most fatal gynecologic cancers and the fifth leading cause of cancer death in women (Ferlay et al., 2013). Each year, approximately 239,000 women are newly diagnosed with ovarian cancer and there are 152,000 deaths, ranking seventh in the incidence rate of female malignant cancers (Reid et al., 2017). In China, it accounted for an estimated 52,000 new cases and 30,000 deaths, and the annual increases in morbidity and mortality were 6.3% and 21.6%, respectively, showing a significant upward trend (Chen et al., 2016). Due to the absence of specific clinical symptoms in the early stages, 70% of ovarian cancer patients are diagnosed in advanced stages.

The current initial therapy for newly diagnosed ovarian cancer is debulking surgery combined with platinum-based chemotherapy/neoadjuvant chemotherapy (Lawrie et al., 2015). However, 70%–80% of advanced patients will experience a relapse within 3 years even after a complete response to primary platinum-based chemotherapy, with a 5-year overall survival rate for only 29% patients (Matulonis et al., 2016; Ledermann et al., 2018; Siegel et al., 2020). The progression-free survival (PFS) of patients with recurrent ovarian cancer gradually shortened with each successive line of treatment, eventually leading to drug resistance and tolerability issues and more difficulty in treatment regimen selection, which seriously affected the survival and quality of life of patients (Hanker et al., 2012). Therefore, it is the common wish of clinicians and patients to delay the recurrence time as much as possible. In recent years, the emergence of poly ADP-ribose polymerase (PARP) inhibitors (PARPis) has made a major breakthrough in the maintenance therapy of ovarian cancer (Ledermann et al., 2014).

PARPis are potentially synthetic lethal effects for the treatment of cancers characterized by specific DNA-repair defects, such as tumor cells that contain BRCA1 and/or BRCA2 (BRCA1/2) mutations and present defects in homologous recombination repair (Bryant et al., 2005; Farmer et al., 2005; Ashworth, 2008). Olaparib, the world’s first PARPi, has been approved by the US Food and Drug Administration (FDA) as maintenance therapy for ovarian cancer patients (Moore et al., 2018). Clinical studies have shown that olaparib significantly prolongs the PFS in BRCA1/2 mutant ovarian cancer patients during both first-line maintenance therapy and platinum-sensitive relapse (PSR) maintenance treatment (Ledermann et al., 2016; Moore et al., 2018). In a clinical trial, SOLO1 demonstrated that olaparib maintenance therapy distinctly prolonged PFS for more than 3 years in advanced ovarian cancer patients with a BRCA1/2 mutation after complete or partial response to first-line platinum-containing chemotherapy compared with placebo (Moore et al., 2018).

In an international, multicenter, double-blind, randomized, placebo-controlled phase 3 trial (SOLO2 trial, NCT01874353), it was found that the median PFS assessed by the investigator in PSR ovarian cancer with germline BRCA1 or BRCA2 mutation (gBRCA) was significantly longer in the olaparib arm at 19.1 months, compared with 5.5 months in the placebo arm. Olaparib reduced the risk of disease progression or death by 70% (HR = 0.30, 95% CI: 0.22–0.41, *p* < 0.0001) (Pujade-Lauraine et al., 2017). Meanwhile, the latest overall survival (OS) results of SOLO2 published in《Lancet oncology》showed that the median OS of the olaparib group was 51.7 months and that of the placebo group was 38.8 months. Olaparib significantly prolonged the OS by more than 1 year compared with placebo and reduced the risk of death by 26% (Poveda et al., 2021). These results indicate that olaparib provides an unprecedented extension of OS in PSR ovarian cancer patients with BRCA1/2 mutation, which is also the first PARPi to have an OS benefit. In China, olaparib was first approved in August 2018 by the National Medical Products Administration (NMPA) for maintenance therapy with PSR ovarian cancer patients who had achieved complete remission (CR) or partial remission (PR) after platinum-containing chemotherapy. Subsequently, in December 2019, NMPA of China approved olaparib for first-line maintenance therapy in newly diagnosed advanced epithelial ovarian cancer patients with BRCA mutations.

However, although olaparib shows obvious advantages in the maintenance therapy for patients with PSR ovarian cancer with BRCA1/2 mutation, its high treatment cost limits its feasibility as a clinical treatment option. The purpose of this study was to evaluate the cost-effectiveness of olaparib vs. placebo as a maintenance therapy in patients with PSR ovarian cancer with BRCA1/2 mutation from a Chinese healthcare perspective.

## Methods

### Model structure

**FIGURE 1:**
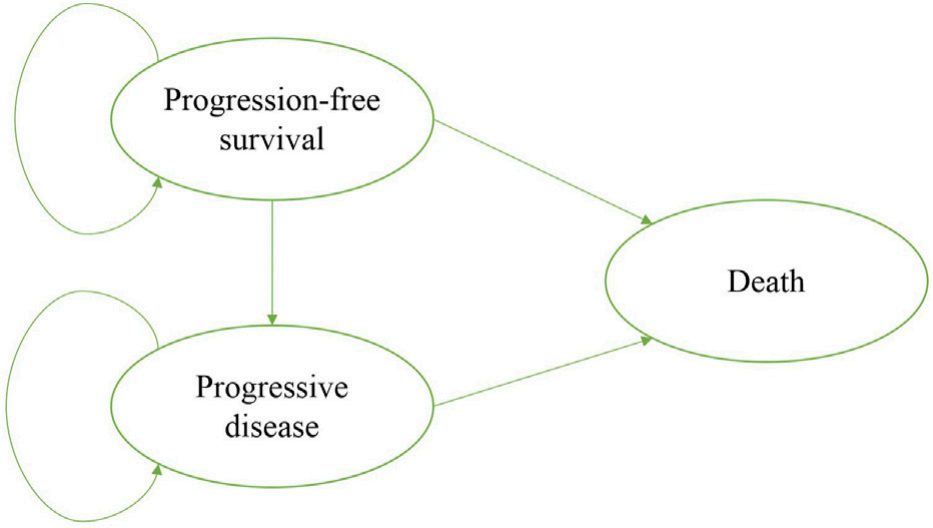
Markov model simulated the three health states: progression-free survival, progressive disease, and death.

A state-transition Markov model was developed to estimate the clinical and economic outcomes of olaparib versus placebo as a maintenance therapy in patients with PSR ovarian cancer and BRCA1/2 mutation. Three mutually exclusive health states: progression-free survival (PFS), progressive disease (PD), and death were included in the model, which reflected the disease course for patients (Figure 1). The initial health state for all patients was PFS, and patients either remained in their assigned health state or progressed to a new health state during each Markov cycle (Wu et al., 2018). The time horizon of the model was 10 year and the Markov cycle length was 1 month in the model. The primary outcomes of the study were quality-adjusted life-years (QALYs) and cost. The future costs and benefits were discounted at an annual rate of 3%, according to the WHO guidelines for pharmacoeconomic evaluations (Murray et al., 2000). All costs had been adjusted to 2020 prices according to the local Consumer Price Index and were presented in US dollars ($1 = ¥6.9). A cost-effectiveness analysis was conducted to evaluate the outcomes of the two strategies and was presented as incremental cost-effectiveness ratios (ICERs). The formula used to calculate the ICER is as follows: ICER = (Cost [Olaparib]-Cost [placebo])/(QALY [Olaparib]-QALY [placebo]). We used 3 × the per capita gross domestic product (GDP) of China in 2020 ($31,498.70) as the willingness-to-pay (WTP) threshold, in line with the WHO recommendations. Model development and outcomes analysis were performed in the TreeAge Pro 2019 software (Williamstown, MA, United States) and R software (version 4.0.5, Vienna, Austria).

### Clinical data

**FIGURE 2:**
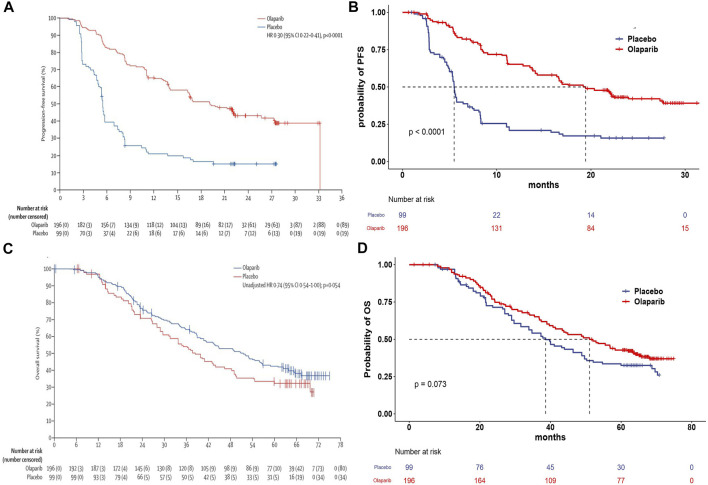
**(A)** Kaplan–Meier curve of progression-free survival from the SOLO2 trial. **(B)** Simulate progression-free survival curve for the olaparib and the placebo group. **(C)** Kaplan–Meier curve of the overall survival from the SOLO2 trial. **(D)** Simulate overall survival curve for the olaparib group and the placebo group. Abbreviations: OS, overall survival; PFS, progression-free survival.

The clinical efficacy and safety data were based on the patients in the SOLO2 trial, a randomized, double-blind, placebo-controlled, multicenter, phase 3 trial (Pujade-Lauraine et al., 2017; Poveda et al., 2021). Eligible patients, with PSR ovarian cancer and BRCA1/2 mutation, were randomized in a 2:1 ratio to receive olaparib maintenance monotherapy (*n* = 196) or matching placebo (*n* = 99). Patients received either olaparib (300 mg in two 150 mg tablets, twice daily) or placebo (twice daily) until disease progression or unacceptable toxicity. The median OS was 51.7 months (95% CI:41.5–59.1) in the olaparib group and 38.8 months (95% CI: 31.4–48.6) in the placebo group. The median PFS was 19.1 months (95% CI:16.3–25.7) in the olaparib group and 5.5 months (95% CI: 5.2–5.8) in the placebo group. The transition probabilities of each health state were estimated from Kaplan–Meier survival curves which were obtained from the SOLO2 trial. In order to get the individual patient data, the Kaplan–Meier curves of PFS and OS for the two groups were extracted by the GetData Graph Digitizer software (Version 2.26), which digitized data points from an image file. To extrapolate the probability of survival beyond the observation period, the Weibull distribution was fitted to the data for PFS and OS curves using R statistical software (version 4.0.5, Vienna, Austria). The estimated scale (λ) and shape (γ) parameters, standard error, and 95% confidence interval were presented in Table 1. The survival probability at time t was calculated using the following formula: S(t) = exp (-λt^γ^), and the transition probability at a given cycle t was calculated using the following formula: P(t) = 1-exp [λ(t-1)^γ^-λt^γ^] (Diaby et al., 2014; Liu et al., 2019). The transition probability from PFS to death state is derived from the natural death rate of the Chinese population in 2020 (0.707%) (National Bureau of Statistics, 2020). The survival curve simulation results are shown in Figure 2.

**TABLE 1:** Weibull parameters of the model estimated for progression-free and overall survival curves.

Group		Parameter	Mean	SE	95% CI	
					Low	Up
Olaparib	PFS	Scale (λ)	0.015753	0.005225	0.008223	0.030178
		Shape (γ)	1.255852	0.104899	1.066202	1.479237
	OS	Scale (λ)	0.001509	0.000779	0.000549	0.041519
		Shape (γ)	1.543080	0126463	1.314099	1.811960
Placebo	PPS	Scale (λ)	0.078114	0.020234	0.047016	0.129781
		Shape (γ)	1.083484	0.093435	0.914994	1.282999
	OS	Scale (λ)	0.002097	0.001341	0.000599	0.007343
		Shape (γ)	1.534778	0158965	1.252801	1.880221

Abbreviations: PFS, progression-free survival; OS, overall survival; SE, standard error; 95% CI, 95% confidence interval.

### Costs and utilities

The cost data were estimated from the perspective of the Chinese healthcare system. The following direct medical cost components were included in the model: the cost of olaparib, BRCA1/2 mutation testing, radiological examinations, management of treatment-related grade 3–4 serious adverse events (SAEs), cost of salvage therapy, routine follow-up, and terminal care at end-of-life (Table 2). Once the disease progressed, salvage chemotherapy was available. To simplify the model, the subsequent therapy was assumed to be paclitaxel and carboplatin on the basis of the NCCN guide. To estimate the dosage of chemotherapeutic agents (Zhang et al., 2020), it was assumed that a typical patient weighed 65 kg and had a height of 1.64 m, resulting in a body surface area (BSA) of 1.72 m^2^. The costs related to SAEs were calculated by multiplying the incidence of the SAEs by the cost of managing the SAEs per event. The most common adverse events, including anemia, neutropenia, nausea, fatigue or asthenia, and the incidence rates of adverse events that occurred in two groups were obtained from the SOLO2 trial. Finally, the cost of BRCA1/2 mutation testing and the cost of terminal care during the final month of life were also included. All costs were derived from local hospitals or previously published studies (Wu et al., 2012; Chen et al., 2020; Li et al., 2020; Zhang et al., 2020; Cheng et al., 2021). Health utility values for each health stage were derived from a recently published study that used the EuroQol five-dimension five-level (EQ-5D-5L) health status questionnaire to assess the quality of life in the SOLO2 trial (Friedlander et al., 2018; Cheng et al., 2021). Disutility due to SAEs was not included in the model as the effect of AEs was assumed to be captured in the utility values collected from the SOLO2 trial. Furthermore, a half-cycle correction was implemented, according to the TreeAge Pro 2019 manual.

**TABLE 2:** Model economic parameters and the range of the sensitivity analysis.

Variables	Base case (rang)	Distribution	Source
Costs ($)			
Olaparib per 150 mg	14.78 (11.82–17.74)	Triangle	Local charge
BRCA1/2 mutation testing	507.25 (405.80–608.70)	Triangle	Local charge
Routine follow-up tests per cycle	35.63 (28.50–42.76)	Triangle	Chen et al. (2020)
Radiological examinations per cycle	64.73 (51.78–77.68)	Triangle	Chen et al. (2020)
Salvage therapy per cycle	270.20 (216.16–324.24)	Triangle	Local charge
Terminal care in end-of-life	2,212.80 (1770.24–2,655.36)	Triangle	Cheng et al. (2021)
Costs of serious adverse events ($)			
Anemia per unit	531.70 (425.36–638.04)	Triangle	Wu et al. (2012)
Neutropenia per unit	530.80 (424.64–636.96)	Triangle	Wu et al. (2012)
Nausea per unit	40.00 (32.00–48.00)	Triangle	Li et al. (2020)
Fatigue or asthenia per unit	110.30 (88.24–132.36)	Triangle	Zhang et al. (2020)
Risks of serious adverse events in Olaparib group (grade 3 or 4) %			
Anemia	19 (15.2–22.8)	Beta	Pujade-Lauraine et al. (2017)
Neutropenia	5 (4–6)	Beta	Pujade-Lauraine et al. (2017)
Nausea	3 (2.3–3.6)	Beta	Pujade-Lauraine et al. (2017)
Fatigue or asthenia	4 (3.2–4.8)	Beta	Pujade-Lauraine et al. (2017)
Risks of serious adverse events in Placebo group (grade 3 or 4) %		
Anemia	2 (1.6–2.4)	Beta	Pujade-Lauraine et al. (2017)
Neutropenia	4 (3.2–4.8)	Beta	Pujade-Lauraine et al. (2017)
Nausea	0 (0–0)	Beta	Pujade-Lauraine et al. (2017)
Fatigue or asthenia	2 (1.6–2.4)	Beta	Pujade-Lauraine et al. (2017)
Utility value			
PFS	0.81 (0.729–0.891)	Beta	(Friedlander et al., 2018; Cheng et al., 2021)
PD	0.74 (0.666–0.814)	Beta	(Friedlander et al., 2018; Cheng et al., 2021)
Body surface area (m^2^)	1.72 (1.38–2.06)	Triangle	Zhang et al. (2020)
Discount rate (%)	3 (0–8)	Fixed in PSA	Murray et al. (2000)

Abbreviations: PFS, progression-free survival; PD, progressive disease; PSA, probabilistic sensitivity analyses.

### Sensitivity analyses

To explore the impact of uncertain model parameters on the outcomes, one-way and probabilistic sensitivity analyses (PSA) were performed in this research. In the one-way sensitivity analysis, relevant parameters were changed one-by-one to their respective upper and lower boundaries, with a range of ± 20% of the base case value, in order to identify the parameters that most significantly influenced the economic outcomes. The results of the one-way sensitivity analysis were plotted in tornado diagrams according to the extent of the parameter’s impact on the ICER. The PSA was performed to assess the effects of uncertainty in all model parameters simultaneously. The model was run 1,000 times through random sampling, in which the parameters were changed with assigned probability distributions (triangle distribution for costs, beta distribution for the probability parameters and utilities). The results of the PSA were presented as a cost-effectiveness acceptability curve and a probabilistic scatter plot, to estimate the WTP threshold for an incremental unit of effectiveness.

## Results

### Base case analysis

Over a 10-year time horizon, the olaparib group gained 3.42 QALYs at a cost of $56,315.60. In the placebo group, the effectiveness was 2.86 QALYs while the cost was $13,022.68. Compared with placebo, the mean incremental effect and cost were 0.56 QALYs and $43,292.92 for the olaparib. The ICER for olaparib versus placebo was $77,620.56/QALY (Table 3). At the Chinese cost-effectiveness WTP threshold of $31,498.70/QALY, olaparib was not a cost-effective treatment strategy compared with placebo.

**TABLE 3:** Cost and outcome results of the cost-effectiveness analysis.

Parameters	Olaparib group	Placebo group
Costs ($)		
PFS state	46,880.78	1,610.31
PD state	9,434.82	11,412.37
Total Cost	56,315.60	13,022.68
Incremental costs ($)	43,292.92	—
Effectiveness (QALYs)		
PFS state	1.66	0.69
PD state	1.76	2.17
Total effectiveness	3.42	2.86
Incremental effectiveness (QALYs)	0.56	—
ICER ($/QALY)	77,620.56	—

Abbreviations: PFS, progression-free survival; PD, progressive disease; QALYs, Quality-adjusted life-years; ICER, incremental cost-effectiveness ratios.

### Sensitivity analyses

**FIGURE 3:**
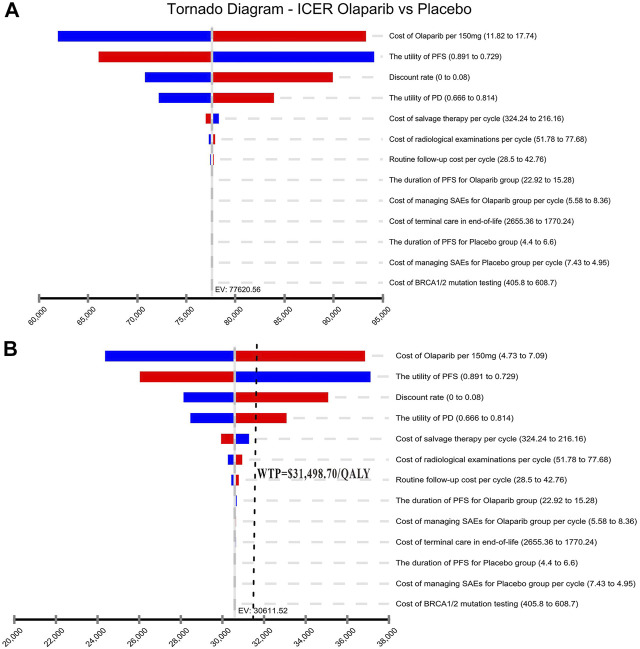
Tornado diagram of one-way sensitivity analysis. It summarized the results of one-way sensitivity analysis, which listed influential parameters in descending order according to their effect on the ICER over the variation of each parameter value. **(A)** The base case analysis. **(B)** 60% discount of olaparib. Abbreviations: ICER, incremental cost-effectiveness ratios; PFS, progression-free survival; PD, progressive disease; SAEs serious adverse events.

**FIGURE 4:**
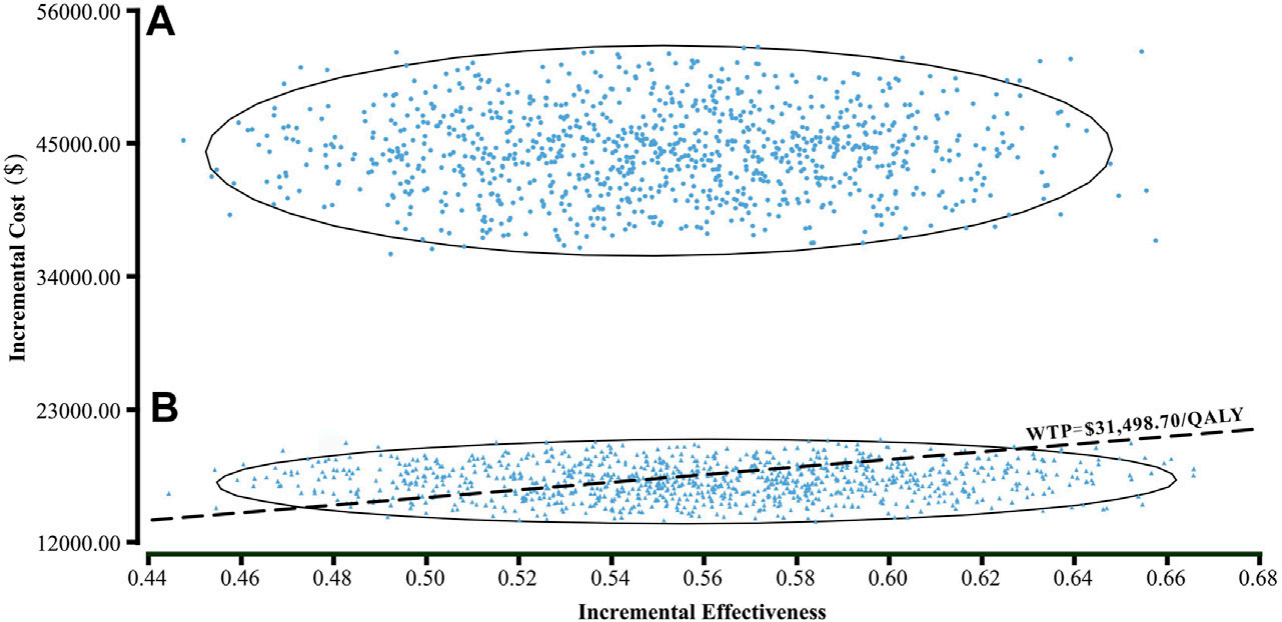
A probabilistic scatter plot of the ICER between the olaparib and placebo group. Each dot represents the ICER for one simulation. An ellipse means 95% confidence interval. Dots that are located below the ICER threshold represent cost-effective simulations. **(A)** The base case analysis. **(B)** 60% discount of olaparib. Abbreviations: WTP willingness-to-pay.

**FIGURE 5:**
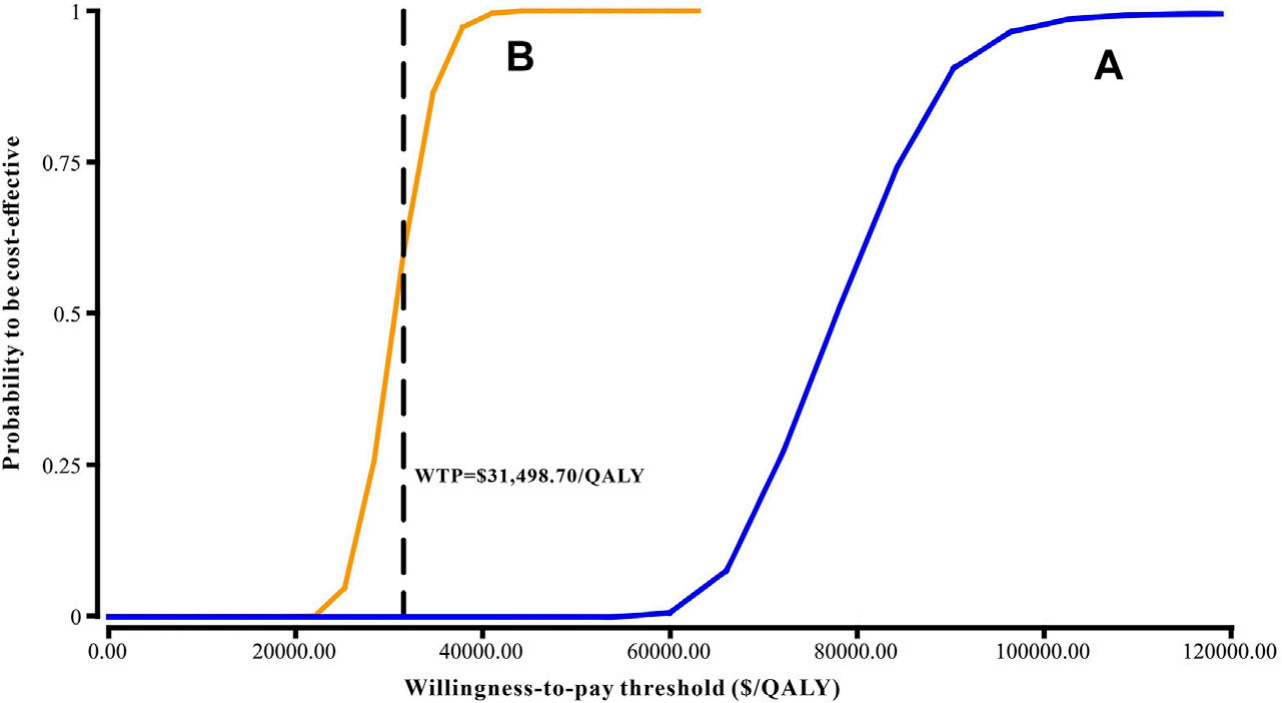
Cost-effectiveness acceptability curve. **(A)** The base case analysis. **(B)** 60% discount of olaparib. Abbreviations: WTP, willingness-to-pay.

Results of the one-way sensitivity analysis were shown with tornado diagrams (Figure 3). The most influential variables were the cost of olaparib per 150 mg and the utility of PFS. However, altering these parameters could not invert the economic outcomes of the model, $61,932.03-$93,309.08/QALY and $66,050.44-$94,105.02/QALY, respectively. Other parameters had a moderate or mild impact on the economic outcomes, and none of the variables could reduce the ICER value below the thresholds. Nevertheless, the ICER could be lower than the threshold in China ($31,498.70) if the cost of olaparib per 150 mg was reduced by 60%, with an ICER of $30,611.52/QALY (Figure 3B). The probabilistic scatter plot and cost-effectiveness acceptability curve were shown in Figures 4, 5, respectively. In the base case analysis, the probabilistic sensitivity analysis demonstrated that there was approximately no cost-effective probability at a threshold of $31,498.70. When the price of olaparib per 150 mg was reduced by 60%, there was a nearly 55% likelihood that olaparib would become cost-effective (Figure 4B). Correspondingly, the acceptability curves showed that the probability of cost-effectiveness also increased with an increase in the WTP threshold, which was sensitive to the thresholds from approximately $60,000 to $120,000 in the base case analysis and from approximately $25,000 to $40,000 in the 60% discount of olaparib (Figure 5B).

## Discussion

Over the past several decades, cytoreductive surgery, chemotherapy, or antiangiogenic agents such as paclitaxel and bevacizumab have been commonly used as therapies for newly diagnosed and relapsed ovarian cancer (Markman et al., 2003; Perren et al., 2011; Gallotta et al., 2019; Gallotta et al., 2020). However, frequent intravenous administration limits their utility, and bevacizumab benefits only in PFS prolonged by 3–4 months (Markman et al., 2003; Perren et al., 2011; Coleman et al., 2017). Recently, PARPis have shown excellent success in delaying the progression of ovarian cancer. Olaparib maintenance treatment significantly prolonged PFS of BRCA mutant ovarian cancer patients with primary treatment and PSR by more than 3 years (56 vs. 13.8 months) and 13.6 months (19.1 vs. 5.5 months), respectively (Pujade-Lauraine et al., 2017; Moore et al., 2018). Olaparib has been recommended in China as maintenance therapy for BRCA mutant and recurrent ovarian cancer in complete or partial response to primary therapy. All previous studies on PARPi took PFS as an evaluation indicator (Ledermann et al., 2012; Moore et al., 2018). In the latest SOLO2 trial, we obtained the world’s first OS data of PARPi used in the treatment of PSR ovarian cancer (Poveda et al., 2021). Exhilaratingly, olaparib as maintenance therapy for PSR ovarian cancer with BRCA mutations significantly extended OS by 12.9 months (51.7 vs. 38.8 months).

To our knowledge, this is the first economic analysis conducted to evaluate the cost-effectiveness of olaparib maintenance therapy versus placebo in PSR ovarian cancer patients with BRCA mutations in China. Our study showed that olaparib achieved an incremental cost of $77,620.56 per QALY compared with placebo, which was far above the Chinese WTP threshold of $31,498.70/QALY, suggesting that olaparib may not be a cost-effective treatment option as a maintenance strategy for PSR ovarian cancer with BRCA mutations. This finding was supported by the one-way sensitivity analysis as variations in model estimates by ± 20% and probabilistic sensitivity analysis.

In the one-way sensitivity analysis, the cost of olaparib per 150 mg and the utility of PFS had the greatest impact on ICER. Other costs in the medical process, including examination and treatment for grade 3 ∼ 4 adverse reactions, had little influence on the results. The probability sensitivity analysis showed that the probability of the ICER value in the olaparib group was lower than the WTP threshold ($31,498.70/QALY) which was 0%. As WTP ranges from $0 to $120,000/QALY, the probability of olaparib being cost-effective increases with the augmentation of WTP. Although the WTP thresholds in different regions have different cost-effectiveness, ICER values in the olaparib group are still above the threshold recommended by rich developed countries, such as £20,000–30,000 per QALY, proposed by the UK’s National Institute for Health and Care Excellence (NICE) (National Institute for Health and Care Excellence, 2013). Moreover, even in the more economically developed regions of China, for example, Beijing (WTP = $72,886.96/QALY), Shanghai (WTP = $69,297.83/QALY), Jiangsu (WTP = $55,341.30/QALY), Fujian (WTP = $48,046.09/QALY) QALY), and Zhejiang (WTP = $48,021.74/QALY), olaparib maintenance therapy is not cost-effective as well. However, olaparib maintenance treatment for PSR ovarian cancer with a BRCA mutation became cost-effective with an ICER ($30,611.52/QALY) value lower than the WTP threshold when the average price of olaparib was reduced by 60% from $14.78 to approximately $5.91 per 150 mg. Additionally, the utility of PFS had a high influence on the model results, but even if the utility of PFS changed from 0.81 to 1, the ICER value ($55,015.13 ∼ 77,620.56 per QALY) was always higher than WTP, which did not lead to the inversion of economic outcomes.

The results of this study are consistent with similar previous studies (Smith et al., 2015). An economic study in the United States showed that for PSR ovarian cancer patients with gBRCA mutation, maintenance therapy with olaparib is also not cost-effective with an ICER of $258,864 per progression-free life-year saved (PF-LYS) (Smith et al., 2015). However, olaparib was cost-effective in the first-line maintenance therapy for BRCA mutant ovarian cancer in both the United States and Singapore (Muston et al., 2020; Tan et al., 2021). From the US third-party payer perspective, the incremental cost per QALY gained for olaparib was $51,986, which had a 52.1% probability of being cost-effective vs. surveillance at a WTP threshold of $50,000 per QALY gained (Muston et al., 2020). In Singapore, olaparib maintenance therapy versus routine surveillance (RS) resulted in an ICER of Singapore dollar (SGD) 19,822 per QALY gained, which had a 87% probability of being cost-effective at a WTP of SGD 60,000 per QALY gained (Tan et al., 2021). Furthermore, ICER values for olaparib as the first-line maintenance therapy were lower than those of second-line or higher maintenance treatment with PSR ovarian cancer. Olaparib might be a more cost-effective strategy for the first-line use than the second-line given a longer PFS benefit as shown in SOLO1 (median PFS gain of approximately 3 years) and SOLO2 (median PFS gain of 13.6 months) (Pujade-Lauraine et al., 2017; Moore et al., 2018). Due to the long PFS with a low risk of recurrence, it may be inappropriate to continue using parametric models after a certain time point. Different parameter models could be used for piecewise fitting, and it is a very good time node when most patients stopped receiving olaparib in a clinical trial. Nevertheless, regardless of molecular signature, currently available first-line maintenance therapies for primary and advanced ovarian cancer, such as olaparib, olaparib-bevacizumab, bevacizumab, and niraparib, are not cost-effective at a WTP threshold of $100,000/PF-LYS in the United States as compared to observations (Penn et al., 2020). Among them, in patients with BRCA mutations, olaparib was considered the most cost-effective. However, for homologous recombination deficient patients without a BRCA mutant, olaparib-bevacizumab was the most cost-effective therapy. Therefore, the FDA has approved a maintenance therapy for a specific molecular subgroup of ovarian cancer. On 19 December 2018, olaparib was used in patients with BRCA mutations, and on 8 May 2020, olaparib combined with bevacizumab was used to treat patients with homologous recombination deficiency (US Food and Drug Administration, 2018; US Food and Drug Administration, 2020). Another clinical study has suggested that assessing germline and somatic BRCA mutation status can help recurrent ovarian cancer patients choose a more appropriate treatment strategy (Gallotta et al., 2019).

From the perspective of the Chinese healthcare system, the therapeutic schedule in our study was derived from RCT and Chinese guidelines. The treatment costs come from local large public hospitals, and the drugs are priced by the National Development and Reform Commission. Currently, Chinese measures to control drug prices mainly include volume-based procurement (VBP) and inclusion in medical insurance (Diao et al., 2021; Yuan et al., 2021). In this model, we take into account, direct costs as much as possible. Our study will provide Chinese policymakers with a reasonable reference price for olaparib maintenance treatment of ovarian cancer.

Similarly, outcomes-based contract (OBC), performance-based agreement (PBA), and risk sharing agreement (RSA) have emerged as promising avenues for payers to improve health outcomes and reduce the risk of cost and finance in Western countries (Navarria et al., 2015; Nazareth et al., 2017; Vreman et al., 2020; Zaric, 2021), even though they are inherently more complex to evaluate and to implement than standard discounts (Bohm et al., 2022). According to the real world data from the European Medicines Agency, it is a feasible method to calculate the probability of death in first-line therapy, the probability of second-line treatment, and the incidence of adverse events (Breccia et al., 2020; Olimpieri et al., 2020; Breccia et al., 2021), which is worthy of reference in the pharmacoeconomic evaluation of various countries.

Our study inevitably had some limitations that warrant discussion. First, the most commonly used two-parameter Weibull survival to extrapolate the tails of survival beyond the follow-up duration of the trial is an inevitable limitation of this study. The survival distributions include Weibull, Log-logistic, Log-normal, Gamma, and Exponential, which may not accurately reflect the real world condition. However, thanks to the good fitness of the model, the model uncertainty surrounding the long-term survival rates is small. Second, our model did not evaluate the impact of different chemotherapies after disease progression, which may not reflect the current Chinese clinical practice situation precisely. However, the result of the sensitivity analysis supported that the costs associated with disease progression did not have an important impact on economic outcomes. Third, we only considered the most common grade 3/4 SAEs in the model. We hypothesized that low-probability adverse events would not change the final conclusions of the study, and the sensitivity analysis showed that the result was not sensitive to SAEs-related parameters. Fourth, utility scores in the study were derived from previously published literature, which might lead to biased model outcomes. Finally, due to the unbalanced economic development in various regions of China, the applicability of this study may be limited. However, because the findings of this evaluation reflected the general clinical conditions of managing patients with PSR ovarian cancer and BRCA1/2 mutation, this study might be a valuable reference for decision makers.

## Conclusion

Olaparib as maintenance therapy for PSR ovarian cancer with BRCA mutations is not considered to be cost effective, compared with placebo from the perspective of the Chinese healthcare system. However, our results support the use of olaparib as maintenance therapy for PSR ovarian cancer patients with a BRCA1/2 mutation as olaparib is not only highly effective but also has strong potential to become a cost-effective treatment option when the cost is reduced by 60% in China. This would significantly improve the outcomes of ovarian cancer patients. Although the results are reported in the setting of the People’s Republic of China, we believe they can easily be generalized to other developing regions and are potentially helpful to healthcare system decision-making.

## Data Availability

The original contributions presented in the study are included in the article/Supplementary Material; further inquiries can be directed to the corresponding author.

## References

[B1] Ashworth A. (2008). A synthetic lethal therapeutic approach: poly(ADP) ribose polymerase inhibitors for the treatment of cancers deficient in DNA double-strand break repair. J. Clin. Oncol. 26 (22), 3785–3790. 10.1200/jco.2008.16.0812 18591545

[B2] Bohm N. Bermingham S. Grimsey Jones F. Gonçalves-Bradley D. C. Diamantopoulos A. Burton J. R. (2022). The challenges of outcomes-based contract implementation for medicines in europe. PharmacoEconomics 40 (1), 13–29. 10.1007/s40273-021-01070-1 34480324 PMC8738500

[B3] Breccia M. Olimpieri P. P. Olimpieri O. Pane F. Iurlo A. Foggi P. (2020). How many chronic myeloid leukemia patients who started a frontline second-generation tyrosine kinase inhibitor have to switch to a second-line treatment? A retrospective analysis from the monitoring registries of the Italian medicines agency (aifa). Cancer Med. 9 (12), 4160–4165. 10.1002/cam4.3071 32319737 PMC7300412

[B4] Breccia M. Celant S. Olimpieri P. P. Olimpieri O. M. Pane F. Iurlo A. (2021). Mortality rate in patients with chronic myeloid leukemia in chronic phase treated with frontline second generation tyrosine kinase inhibitors: A retrospective analysis by the monitoring registries of the Italian medicines agency (AIFA). Ann. Hematol. 100 (2), 481–485. 10.1007/s00277-021-04406-1 33415425

[B5] Bryant H. E. Schultz N. Thomas H. D. Parker K. M. Flower D. Lopez E. (2005). Specific killing of BRCA2-deficient tumours with inhibitors of poly(ADP-ribose) polymerase. Nature 434 (7035), 913–917. 10.1038/nature03443 15829966

[B6] Chen W. Zheng R. Baade P. D. Zhang S. Zeng H. Bray F. (2016). Cancer statistics in China, 2015. Ca. Cancer J. Clin. 66 (2), 115–132. 10.3322/caac.21338 26808342

[B7] Chen Z. Tian F. Xu T. (2020). Cost-effectiveness analysis of bevacizumab combined with standard chemotherapy in patients with recurrent ovarian cancer. Chin. J. Hosp. Pharm. 40 (2), 189–193.

[B8] Cheng L. J. Wong G. Chay W. Y. Ngeow J. Tan Y. Soon S. S. (2021). Cost-effectiveness of olaparib maintenance therapy when used with and without restriction by BRCA1/2 mutation status for platinum-sensitive relapsed ovarian cancer. Expert Rev. pharmacoecon. Outcomes Res. 21 (3), 441–448. 10.1080/14737167.2021.1890587 33593205

[B9] Coleman R. L. Brady M. F. Herzog T. J. Sabbatini P. Armstrong D. K. Walker J. L. (2017). Bevacizumab and paclitaxel-carboplatin chemotherapy and secondary cytoreduction in recurrent, platinum-sensitive ovarian cancer (NRG oncology/gynecologic oncology group study GOG-0213): A multicentre, open-label, randomised, phase 3 trial. Lancet. Oncol. 18 (6), 779–791. 10.1016/s1470-2045(17)30279-6 28438473 PMC5715461

[B10] Diaby V. Adunlin G. Montero A. J. (2014). Survival modeling for the estimation of transition probabilities in model-based economic evaluations in the absence of individual patient data: A tutorial. PharmacoEconomics 32 (2), 101–108. 10.1007/s40273-013-0123-9 24338265

[B11] Diao Y. Lin M. Xu K. Huang J. Wu X. Li M. (2021). How government health insurance coverage of novel anti-cancer medicines benefited patients in China - a retrospective analysis of hospital clinical data. BMC Health Serv. Res. 21 (1), 856. 10.1186/s12913-021-06840-3 34419013 PMC8380313

[B12] Farmer H. McCabe N. Lord C. J. Tutt A. N. Johnson D. A. Richardson T. B. (2005). Targeting the DNA repair defect in BRCA mutant cells as a therapeutic strategy. Nature 434 (7035), 917–921. 10.1038/nature03445 15829967

[B13] Ferlay J. Steliarova-Foucher E. Lortet-Tieulent J. Rosso S. Coebergh J. W. Comber H. (2013)., 49. Oxford, England, 1374–1403. 10.1016/j.ejca.2012.12.027 Cancer incidence and mortality patterns in europe: Estimates for 40 countries in 2012 Eur. J. Cancer 6 23485231

[B14] Friedlander M. Gebski V. Gibbs E. Davies L. Bloomfield R. Hilpert F. (2018). Health-related quality of life and patient-centred outcomes with olaparib maintenance after chemotherapy in patients with platinum-sensitive, relapsed ovarian cancer and a BRCA1/2 mutation (SOLO2/ENGOT ov-21): A placebo-controlled, phase 3 randomised trial. Lancet. Oncol. 19 (8), 1126–1134. 10.1016/s1470-2045(18)30343-7 30026002 PMC7869962

[B15] Gallotta V. Conte C. D'Indinosante M. Capoluongo E. Minucci A. De Rose A. M. (2019). Prognostic factors value of germline and somatic brca in patients undergoing surgery for recurrent ovarian cancer with liver metastases. Eur. J. Surg. Oncol. 45 (11), 2096–2102. 10.1016/j.ejso.2019.06.023 31227342

[B16] Gallotta V. Bruno M. Conte C. Giudice M. T. Davià F. Moro F. (2020). Salvage lymphadenectomy in recurrent ovarian cancer patients: Analysis of clinical outcome and BRCA1/2 gene mutational status. Eur. J. Surg. Oncol. 46 (7), 1327–1333. 10.1016/j.ejso.2020.01.035 32085925

[B17] Hanker L. C. Loibl S. Burchardi N. Pfisterer J. Meier W. Pujade-Lauraine E. (2012). The impact of second to sixth line therapy on survival of relapsed ovarian cancer after primary taxane/platinum-based therapy. Ann. Oncol. 23 (10), 2605–2612. 10.1093/annonc/mds203 22910840

[B18] Lawrie T. A. Winter-Roach B. A. Heus P. Kitchener H. C. (2015). Adjuvant (post-surgery) chemotherapy for early stage epithelial ovarian cancer. Cochrane Database Syst. Rev. 2015 (12), Cd004706. 10.1002/14651858.CD004706.pub5 PMC6457737 26676202

[B19] Ledermann J. Harter P. Gourley C. Friedlander M. Vergote I. Rustin G. (2012). Olaparib maintenance therapy in platinum-sensitive relapsed ovarian cancer. N. Engl. J. Med. 366 (15), 1382–1392. 10.1056/NEJMoa1105535 22452356

[B20] Ledermann J. Harter P. Gourley C. Friedlander M. Vergote I. Rustin G. (2014). Olaparib maintenance therapy in patients with platinum-sensitive relapsed serous ovarian cancer: A preplanned retrospective analysis of outcomes by BRCA status in a randomised phase 2 trial. Lancet. Oncol. 15 (8), 852–861. 10.1016/s1470-2045(14)70228-1 24882434

[B21] Ledermann J. A. Harter P. Gourley C. Friedlander M. Vergote I. Rustin G. (2016). Overall survival in patients with platinum-sensitive recurrent serous ovarian cancer receiving olaparib maintenance monotherapy: An updated analysis from a randomised, placebo-controlled, double-blind, phase 2 trial. Lancet. Oncol. 17 (11), 1579–1589. 10.1016/s1470-2045(16)30376-x 27617661

[B22] Ledermann J. A. Raja F. A. Fotopoulou C. Gonzalez-Martin A. Colombo N. Sessa C. (2018). Corrections to "newly diagnosed and relapsed epithelial ovarian carcinoma: ESMO clinical practice guidelines for diagnosis, treatment and follow-up". Ann. Oncol. 29 (4), iv259. 10.1093/annonc/mdy157 30285216

[B23] Li S. Peng L. Tan C. Zeng X. Wan X. Luo X. (2020). Cost-Effectiveness of ramucirumab plus paclitaxel as a second-line therapy for advanced gastric or gastro-oesophageal cancer in China. PloS one 15 (5), e0232240. 10.1371/journal.pone.0232240 32379763 PMC7205241

[B24] Liu M. Zhang L. Huang Q. Li N. Zheng B. Cai H. (2019). Cost-effectiveness analysis of ceritinib and alectinib versus crizotinib in the treatment of anaplastic lymphoma kinase-positive advanced non-small cell lung cancer. Cancer Manag. Res. 11, 9195–9202. 10.2147/cmar.S223441 31749634 PMC6818540

[B25] Markman M. Liu P. Y. Wilczynski S. Monk B. Copeland L. J. Alvarez R. D. (2003). Phase III randomized trial of 12 versus 3 months of maintenance paclitaxel in patients with advanced ovarian cancer after complete response to platinum and paclitaxel-based chemotherapy: A southwest oncology group and gynecologic oncology group trial. J. Clin. Oncol. 21 (13), 2460–2465. 10.1200/jco.2003.07.013 12829663

[B26] Matulonis U. A. Penson R. T. Domchek S. M. Kaufman B. Shapira-Frommer R. Audeh M. W. (2016). Olaparib monotherapy in patients with advanced relapsed ovarian cancer and a germline BRCA1/2 mutation: A multistudy analysis of response rates and safety. Ann. Oncol. 27 (6), 1013–1019. 10.1093/annonc/mdw133 26961146

[B27] Moore K. Colombo N. Scambia G. Kim B. G. Oaknin A. Friedlander M. (2018). Maintenance olaparib in patients with newly diagnosed advanced ovarian cancer. N. Engl. J. Med. 379 (26), 2495–2505. 10.1056/NEJMoa1810858 30345884

[B28] Murray C. J. Evans D. B. Acharya A. Baltussen R. M. Development of WHO guidelines on generalized cost-effectiveness analysis. Health Econ. (2000) 9(3):235–251. 10.1002/(sici)1099-1050(200004)9:3<235::aid-hec502>3.0.co;2-o 10790702

[B29] Muston D. Hettle R. Monberg M. McLaurin K. K. Gao W. Swallow E. (2020). Cost-effectiveness of olaparib as a maintenance treatment for women with newly diagnosed advanced ovarian cancer and BRCA1/2 mutations in the United States. Gynecol. Oncol. 159 (2), 491–497. 10.1016/j.ygyno.2020.08.013 32951894

[B30] National Bureau of Statistics. National data of national Bureau of statistics in 2020 (2020), Available from: https://data.stats.gov.cn/tablequery.htm?code=AD02.

[B31] National Institute for Health and Care Excellence (2013). Guide to the methods of technological appraisal. London: NICE. 27905712

[B32] Navarria A. Drago V. Gozzo L. Longo L. Mansueto S. Pignataro G. (2015). Do the current performance-based schemes in Italy really work? "Success fee": A novel measure for cost-containment of drug expenditure. Value Health 18 (1), 131–136. 10.1016/j.jval.2014.09.007 25595244

[B33] Nazareth T. Ko J. J. Sasane R. Frois C. Carpenter S. Demean S. (2017). Outcomes-based contracting experience: Research findings from U.S. And European stakeholders. J. Manag. Care Spec. Pharm. 23 (10), 1018–1026. 10.18553/jmcp.2017.23.10.1018 28944734 PMC10398222

[B34] Olimpieri P. P. Di Lenarda A. Mammarella F. Gozzo L. Cirilli A. Cuomo M. (2020). Non-vitamin K antagonist oral anticoagulation agents in patients with atrial fibrillation: Insights from Italian monitoring registries. Int. J. Cardiol. Heart Vasc. 26, 100465. 10.1016/j.ijcha.2019.100465 32021902 PMC6994529

[B35] Penn C. A. Wong M. S. Walsh C. S. (2020). Cost-effectiveness of maintenance therapy based on molecular classification following treatment of primary epithelial ovarian cancer in the United States. JAMA Netw. Open 3 (12), e2028620. 10.1001/jamanetworkopen.2020.28620 33295974 PMC7726632

[B36] Perren T. J. Swart A. M. Pfisterer J. Ledermann J. A. Pujade-Lauraine E. Kristensen G. (2011). A phase 3 trial of bevacizumab in ovarian cancer. N. Engl. J. Med. 365 (26), 2484–2496. 10.1056/NEJMoa1103799 22204725

[B37] Poveda A. Floquet A. Ledermann J. A. Asher R. Penson R. T. Oza A. M. (2021). Olaparib tablets as maintenance therapy in patients with platinum-sensitive relapsed ovarian cancer and a BRCA1/2 mutation (SOLO2/ENGOT-Ov21): A final analysis of a double-blind, randomised, placebo-controlled, phase 3 trial. Lancet. Oncol. 22 (5), 620–631. 10.1016/s1470-2045(21)00073-5 33743851

[B38] Pujade-Lauraine E. Ledermann J. A. Selle F. Gebski V. Penson R. T. Oza A. M. (2017). Olaparib tablets as maintenance therapy in patients with platinum-sensitive, relapsed ovarian cancer and a BRCA1/2 mutation (SOLO2/ENGOT-Ov21): A double-blind, randomised, placebo-controlled, phase 3 trial. Lancet. Oncol. 18 (9), 1274–1284. 10.1016/s1470-2045(17)30469-2 28754483

[B39] Reid B. M. Permuth J. B. Sellers T. A. (2017). Epidemiology of ovarian cancer: A review. Cancer Biol. Med. 14 (1), 9–32. 10.20892/j.issn.2095-3941.2016.0084 28443200 PMC5365187

[B40] Siegel R. L. Miller K. D. Jemal A. (2020). Cancer statistics, 2020. Ca. Cancer J. Clin. 70 (1), 7–30. 10.3322/caac.21590 31912902

[B41] Smith H. J. Walters Haygood C. L. Arend R. C. Leath C. A. 3rd Straughn J. M. Jr (2015). PARP inhibitor maintenance therapy for patients with platinum-sensitive recurrent ovarian cancer: A cost-effectiveness analysis. Gynecol. Oncol. 139 (1), 59–62. 10.1016/j.ygyno.2015.08.013 26303225

[B42] Tan D. S. Chan J. J. Hettle R. Ghosh W. Viswambaram A. Yu C. C. (2021). Cost-effectiveness of olaparib versus routine surveillance in the maintenance setting for patients with BRCA-mutated advanced ovarian cancer after response to first-line platinum-based chemotherapy in Singapore. J. Gynecol. Oncol. 32 (2), e27. 10.3802/jgo.2021.32.e27 33559410 PMC7930440

[B43] Us Food and Drug Administration. FDA approved olapari (LYNPARZA, AstraZeneca Pharmaceuticals LP) for the maintenance treatment of adult patients with deleterious or suspected deleterious germline or somatic BRCA-mutated (gBRCAm or sBRCAm) advanced epithelial ovarian, fallopian tube or primary peritoneal cancer who are in complete or partial response to first-line platinum-based. Published December 26, 2018. Accessed June 9, 2020. Available at: https://www.fda.gov/drugs/fda-approved-olaparib-lynparza-astrazeneca-pharmaceuticals-lp-maintenance-treatment-adult-patients .

[B44] Us Food and Drug Administration. FDA approves olaparib plus bevacizumab as maintenance treatment for ovarian, fallopian tube, or primary peritoneal cancers. Published May 11, 2020. Accessed June 9, 2020. Available at: https://www.fda.gov/drugs/drug-approvals-and-databases/fda-approves-olaparib-plus-bevacizumab-maintenance-treatment-ovarian-fallopian-tube-or-primary .

[B45] Vreman R. A. Broekhoff T. F. Leufkens H. G. Mantel-Teeuwisse A. K. Goettsch W. G. (2020). Application of managed entry agreements for innovative therapies in different settings and combinations: A feasibility analysis. Int. J. Environ. Res. Public Health 17 (22), E8309. 10.3390/ijerph17228309 33182732 PMC7698033

[B46] Wu B. Ye M. Chen H. Shen J. F. (2012). Costs of trastuzumab in combination with chemotherapy for HER2-positive advanced gastric or gastroesophageal junction cancer: An economic evaluation in the Chinese context. Clin. Ther. 34 (2), 468–479. 10.1016/j.clinthera.2012.01.012 22325735

[B47] Wu B. Gu X. Zhang Q. (2018). Cost-effectiveness of osimertinib for EGFR mutation-positive non-small cell lung cancer after progression following first-line EGFR TKI therapy. J. Thorac. Oncol. 13 (2), 184–193. 10.1016/j.jtho.2017.10.012 29101057

[B48] Yuan J. Lu Z. K. Xiong X. Jiang B. (2021). Lowering drug prices and enhancing pharmaceutical affordability: An analysis of the national volume-based procurement (NVBP) effect in China. BMJ Glob. Health 6 (9), e005519. 10.1136/bmjgh-2021-005519 PMC8438819 34518200

[B49] Zaric G. S. (2021). How risky is that risk sharing agreement? Mean-variance tradeoffs and unintended consequences of six common risk sharing agreements. MDM Policy Pract. 6 (1), 1. 10.1177/2381468321990404 PMC7876771 33623819

[B50] Zhang P. F. Xie D. Li Q. (2020). Cost-effectiveness analysis of nivolumab in the second-line treatment for advanced esophageal squamous cell carcinoma. Future Oncol. 16 (17), 1189–1198. 10.2217/fon-2019-0821 32407173

